# Leadership behaviours in interprofessional student teamwork

**DOI:** 10.1186/s12909-022-03923-5

**Published:** 2022-12-02

**Authors:** Christie van Diggele, Chris Roberts, Stuart Lane

**Affiliations:** 1 Faculty of Medicine and Health, The University of Sydney, NSW 2006 Sydney, Australia; 2 Faculty of Medicine and Health, Sydney Medical School, The University of Sydney, Sydney, NSW Australia

**Keywords:** Interprofessional, Leadership, Teamwork, Health professions, Education, Peer review

## Abstract

**Background:**

Effective leaders support high-quality patient care and improve patient safety by embodying a collective leadership style. Training in leadership skills needs to be integrated longitudinally throughout a clinician’s career. Models of leadership drawn from organisational theories can provide a conceptual framework for cultivating student leadership qualities during teamwork and the evaluation of emergent outcomes. Using the conceptual framework of Situational Leadership Theory, we sought to explore the leadership qualities identified by students of their team members, during a large scale interprofessional learning activity.

**Methods:**

In 2018, 1674 students from 11 health disciplines were required to participate in the “Health Collaboration Challenge” (HCC). The HCC required students to work in small interprofessional teams of five or six students. Following team activities, students were required to provide constructive written feedback to their team members. Peer feedback data were coded and categorised into themes using the conceptual framework of Situational Leadership Theory. Data were then quantified within each theme.

**Results:**

A total of 1282 comments were analysed. The most frequent comments related to ‘delegating’ (456/1282, 36%) and ‘supporting’ (402/1282, 31%). This was followed by comments categorised as ‘directing’ (244/1282, 19%), and ‘coaching’ (180/1282, 14%) leadership styles. Notably, a total of 1112/2597 (43%) of comments were unconstructive. A total of 298 comments provided by students informed their peers of areas for self-improvement. The most frequent comments were recommendations relating to ‘active team member contribution’ (111/298; 37%), followed by ‘communication’ (83/298; 28%), ‘interprofessional practice’ (77/298; 26%), and ‘disciplinary knowledge’ (27/298; 9%).

**Conclusion:**

Although most students demonstrated a reasonable ability to display leadership behaviours appropriate to teamwork, further development is needed through training. Leadership skills are an expectation of health professional graduates, and should be explicitly taught and vertically integrated within interprofessional education curricula. Further research is warranted in how students contribute to and understand the requirements of leadership within interprofessional teams.

## Introduction

The impact of COVID-19 exposed existing vulnerabilities within healthcare systems and highlighted the need for educational systems to prepare students for an era of rapid change and constant evolution as new models of care arise [1]. Collaboration within and between health service delivery teams enables accomplishment of common goals to improve patient safety and quality of care [2–5]. High-quality patient care is supported by embodying a collective leadership style [6, 7]. However, there is a shortage of emerging leaders moving into leadership roles, and expectations that a large number of experienced clinicians will retire in the near future [6, 8, 9]. A recent review of health professional leadership programs found notable gaps in the integration of non-physicians with physicians, and limited interactive learning and feedback [10].

Leadership has had a major influence in shaping organisational culture, however, little attention has been paid to the systematic preparation of health professional students as leaders in the various levels of healthcare delivery [6]. It is important to consider how the cultivation of leadership skills can be nurtured and supported within the interprofessional education setting [4, 6, 11, 12]. There is a gap in empirical research exploring the contexts and activities that promote leadership development in interprofessional settings, and how students give and receive peer feedback around leadership qualities. An opportunity to explore these issues arose in the context of a large scale interprofessional learning activity at an Australian university.

Organisational theories assist in understanding student team function and leadership within an educational context. Leadership is socially constructed, and there are many different leadership models [13]. One theoretical perspective suitable for examining team function behaviours in relation to large scale interprofessional activities is Situational Leadership Theory. This theory proposes that leadership is contextual and influenced by situational factors. It suggests that a leader must adjust the degree to which they direct or support their subordinates based on context [14]. Individuals should adapt their leadership style based on the skills of their team members, catering to situational demands [15]. Situational leadership requires individuals to be flexible, assess the situation and adopt a leadership style that best fits the needs of team members. It is based on two key behavioural characteristics: task behaviour (the extent to which responsibilities are assigned); and relational behaviour (the depth to which communication is extended). Situational Leadership Theory posits that there are four styles of leadership [15]: (1) Directing, characterised by ‘high task and low relationship’ behaviours; (2) Delegating, characterised by ‘low relationship and low task’ behaviours; (3) Supporting, characterised by ‘high relationship and low task’ behaviours; and (4) Coaching, characterised by ‘high task and high relationship’ behaviours.

Using Situational Leadership Theory, we sought to explore the leadership qualities identified by students of their team members, during a large scale interprofessional learning activity. Our research question was: What leadership qualities do health professional students display in an interprofessional team setting when working on a shared task?

## Methods

### Participants

In 2018, 1674 students from 11 health disciplines were required to participate in the “Health Collaboration Challenge” (HCC), which has been previously described [16]. Students were from 11 disciplines: Dentistry, Diagnostic radiography, Dietetics, Exercise physiology, Medicine, Nursing, Occupational therapy, Oral health, Pharmacy, Physiotherapy, and Speech pathology.

### Research context

The HCC required students to work in small interprofessional teams of five or six students, each consisting of four or more disciplines. Students completed three team activities based on the review of a complex patient case: (1) develop a one-page patient management plan, (2) produce a five-minute video demonstrating case management, and (3) provide a peer review for two other team videos. The activities were student-led and involved meeting in their assigned teams for a pre-assigned one-day session, with the requirement of completing all components over a six-day period. The tasks were assessable and written feedback was provided to student teams on their performance.

#### Peer review activity

Following completion of the three team activities, students were required to provide written feedback to their own team members, based on their team contributions. The peer review was designed to promote professional behaviours within teams and to develop skills in feedback. Students were required to complete the peer review using the online tool Sparkplus [17]. This tool supports group work by enabling students to self review and peer review via a website, promoting collaborative learning. They were required to:

- Self-assess their own contributions to the activity. Nine statements were provided, and students were required to respond, using a Likert scale of 1 to 5 (1 = strongly disagree; 5 = strongly agree).
- Rate all team members on their contributions to the activity, using the same scale.
- Provide constructive written feedback to at least two team members of their choice on their contributions to the activity. It was noted that feedback should be honest and constructive. An example of peer feedback was provided to students. The feedback was anonymous.

### Study design

#### Data collection and analysis

The focus of this study was on the qualitative (written) feedback provided by students during the peer review activity. Framework analysis was used to code and categorise the data into themes using the conceptual framework of Situational Leadership Theory [18]. All researchers (CvD, SL, CR) participated in an initial calibration exercise where each researcher independently coded and categorised the same 50 instances of students’ peer review comments. Researchers met to discuss any discrepancies in their coding. The first author (CvD) then coded the remaining data into themes. The researchers then quantified the data to show the prevalence of each theme [19]. Feedback of 15 words or less was categorised as being insufficient for analysis. Comments that were cut and pasted were considered unconstructive, so were excluded from data analysis. Data were categorised by the four themes of Situational Leadership Theory:

- *Directing*: is characterised by ‘high task and low relationship’ behaviours. The leader makes decisions and allocates tasks to members, with limited feedback provided to team members. Communication is limited and focused on task or goal achievement.
- *Delegating*: is characterised by ‘low relationship and low task’ behaviour. The leader facilitates decision making by the team, giving team members responsibility for their own tasks. The leader refrains from intervening unless needed, and communication takes place only when required.
- *Supporting*: is characterised by ‘high relationship and low task’ behaviours. Team members are supported by the leader who encourages communication and feedback, with less of a focus being placed on the tasks themselves.
- *Coaching*: is characterised by ‘high task and high relationship’ behaviours. Contribution is encouraged from team members in a cooperative and democratic manner. The leader role models, encouraging communication and feedback. Sometimes knowledge and skills are shared between team members.

During the secondary analysis, the researchers recognised patterns in relation to students providing their peers with suggestions for improvement in future practice. These comments were analysed and coded thematically within descriptive categories. The comments within each category were then quantified. Four themes were used to categorise the data:

- *Communication*: Communication with the team needed to be clearer, conscience, or students needed to ‘speak up’ more.
- *Disciplinary knowledge*: A student’s own sharing of disciplinary knowledge was limited, or they did not know much about the other disciplines they were working with.
- *Interprofessional practice*: Students needed to listen to others, work collaboratively with team members or allow for input from the patient/client.
- *Active team member contributions*: Students needed to be more active team members, contributing more to the task or have more confidence in being actively involved.

### Ethics approval

The University of Sydney Human Research Ethics Committee approved the study (Project number: 2015/556).

## Results

A total of 2597 comments were submitted by students and ranged in length from 1- 212 words per comment. Of these, 1112/2597 (43%) comments were excluded from the study due to being unconstructive comments that consisted of 15 words or less, or duplicate comments that were cut and pasted to multiple team members. Of the remaining 1282 comments coded, the most frequent comments related to the theme of ‘delegating’ (456/1282, 36%), and ‘supporting’ (402/1282, 31%). This was followed by comments categorised as ‘directing’ (244/1282, 19%), and ‘coaching’ (180/1282, 14%). These comments were categorised into four themes and presented in Table 1.

**Table 1:** Feedback from students according to the themes of ‘delegating’, ‘supporting’, ‘directing’ and ‘coaching’ within Situational Leadership Theory

Theme	Examples of student peer feedback comments	No. of similar comments
**Delegating** Details relating to a Delegating leader were linked to shared decision making within the team, and members taking responsibility for their own tasks. Communication was likely to only take place when needed.	*(Name) actively participated in the video and abstract. Added in ideas that we forgot.* *You have contributed well on the day of the group activity. However, ongoing feedback and effort to contribute and assist with the completion of the group project would have been ideal.*	(456/1282, 36%)
**Supporting** Comments categorised as Supporting were those that indicated team communication and feedback, with less of a focus being placed on tasks.	*(Name) was a good team member who put forth her own ideas and listened well to others. She collaborated well with all other disciplines and was able to make recommendations that fit within the contributions of other team members.* *(Name) actively participated and was easy to work with. She contributed to the knowledge of other professional roles as well as her own. She assisted with coming up with different ideas for videos, and was able to make adjustments where needed given the limited resources. Listened and showed respect to others’ opinions.*	(402/1282, 31%)
**Directing** Feedback categorised as Directing indicated that the student receiving the peer feedback made decisions with limited input from others and communication. These comments were mostly focused on task-based items.	*(Name) took the initiative to edit and submit the abstract on our behalf and he edited the document to a professional standard. He applied his clinical knowledge professionally by considering the patient holistically. He was very prompt on communicating the status of editing.* *I appreciate all the hard work you put into the project, but I also feel like you commandeered the abstract and didn’t allow time for appropriate review and excluded some people’s input.*	(244/1282, 19%)
**Coaching** Comments categorised as Coaching described team contribution and cooperative team function, valuing communication, feedback and the sharing of knowledge.	*(Name) is a valuable member of the team. He takes the initiatives and drives our team forward. He would complete his tasks effectively and to a high quality. He’d always be open to actively listen and take in the opinions of other team members, making sure that everyone’s opinions are valued*. *(Name) strongly contributed to the team with his medical knowledge of the patient. He worked collaboratively with others and contributed to new ideas to the team. He displayed a willingness to work to learn other disciplines roles. He demonstrated strong initiative to guide the team as he generated new ideas without prompting. He related to others in an open, friendly and professional manner and showed understanding to other member ideas on how to create the video. He demonstrated leadership skills to keep the team informed with all necessary information. He set goals and allocated times to complete the video and abstract*.	(180/1282, 14%)

A total of 298 comments provided by students informed their peers of areas for self-improvement. These comments were grouped into four themes and are summarised in Table 2. The most frequent comments related to recommendations relating to ‘active team member contribution’ (111/298; 37%), followed by ‘communication’ (83/298; 28%), ‘interprofessional practice’ (77/298; 26%), and ‘disciplinary knowledge’ (27/298; 9%).

**Table 2:** Areas of improvement identified by students in their feedback (*N* = 298)

Category	Examples of student comments	Number of similar comments
**Active team member contribution** *Students needed to be more active team members, contribute more to the task, or have more confidence to be actively involved.*	*…In the future, I hope you have more confidence to share your expertise spontaneously, even amid a team of such forceful personalities as ours.* *Focus more on the purpose of the task, i.e. know what the patient currently already has in place as part of their plan and work around that.*	111/298 (37%)
**Communication** *Communication needed to be clearer, conscience, or students needed to ‘speak up’ more.*	*…Little improvement is suggested, although as per other students, it is recommended that lay terms can be utilised when speaking with other disciplines so a greater understanding can be made by everyone.* *…The only place for improvement I could see with (name) is with his communication style. He is rather quiet and at times can be difficult to understand. In the future (name) might improve his professional competency by being more assertive and being a bit clearer with his language.*	83/298 (28%)
**Interprofessional practice** *Students needed to listen to others, work collaboratively with team members or allow for input from the patient/client.*	*… However, a more holistic management plan could have been delivered by considering how your discipline can collaborate with other discipline, in order to maximise the patient and management goals.* *(Name) was a helpful member of the team and considered her role in helping the client achieve each of her goals. I think something we could all improve on is to address the patient’s concerns and emotional impact in the video.*	77/298 (26%)
**Disciplinary knowledge** *Own disciplinary knowledge sharing was limited, or they did not know much about other disciplines there were working with.*	*… However, I would improve on the knowledge of how dietitians could liaise with speech pathologists, occupational therapists, and physiotherapists. That being said, your knowledge of your own and other disciplines were already very good. Well done!* *…However, I would have liked if you could clearly explain the role of a pharmacist in Lulu’s case (although I probably should have asked). Thanks!*	27/298 (9%)

## Discussion

We sought to explore the leadership qualities and styles identified by students during a large scale, small team interprofessional learning activity, using Situational Leadership Theory as a conceptual framework [15]. The most common leadership styles identified were ‘Delegating’ (36%) and ‘Supporting’ (31%). Fewer responses were identified as ‘Directing’ (19%), and ‘Coaching’ (14%). The need for improvement identified by students included the need for more active contributions to teamwork and clearer communication, including listening to others, demonstrating a greater awareness of interprofessional patient-centred care, and contributing to teamwork by sharing one’s own disciplinary knowledge.

A key task of leadership is utilising appropriate skills and adapting an appropriate style for the given situation in supporting effective team function [20]. Peer feedback provided during team activities, suggests that some students performed their own tasks accurately and efficiently using task-oriented leadership behaviours. Students using relational-oriented behaviours focused on communicating support and appreciation for others. Both types of behaviours are core to basic leadership and are learned behaviours. Behaviours traditionally associated with leadership, such as ‘Directing’ were not always perceived favourably by peers. In line with current literature, student feedback to peers highlighted the importance of listening to others, and considering all viewpoints. Oates (2012) suggests that a key characteristics of tomorrow’s clinical leaders is “being a team player as well as a team leader” [4]. Good team leaders value the opinions of others, and display respectful communication, acknowledging the strengths and ideas of others [4]. Yet if some team members in the group are less confident, capable or willing, a ‘directing’ or ‘coaching’ approach may be appropriate [15, 21]. For example, ‘directing’ will be appropriate in their future workplace context, such as during patient treatment and management during a medical emergency.

A collective leadership style is essential to support excellence in patient care [22]. Our findings align with literature emphasising the importance of cultivating clinical leaders with qualities that include clear and concise communication; the sharing of disciplinary knowledge and willingness to learn from others; collaborative interprofessional practice, whereby team members are encouraged to contribute, and support input from the patient; and active contribution from all team members towards the task and team discussion [4]. A recent systematic review by Sfantou and colleagues (2017) identified a correlation between effective leadership and patient outcomes, finding that effective leadership fosters a high-quality work environment leading to positive patient outcomes, while failure to create a quality workplace ultimately harms patients [23].

While some attempts have been made within the university sector to embed leadership in health professional curricula, there is an identified need for explicit training and development in this area [24]. Rather than being taught informally, skills such as communication and teamwork should be identified as leadership competencies and reinforced throughout a vertically integrated, interprofessional curricula. Steps could be taken to make leadership behaviours more explicit in practice, by creating an awareness of the importance of leadership and how their work environment (and clinical placement) is influenced by good leadership. Leadership in team settings should be specifically identified, trained, rewarded, and encouraged at all levels of a health professional students’ degree.

West et al. (2015) suggest longitudinal leadership development is essential, noting shared and collaborative leadership to be the most effective [22]. Our results indicate that while most students contributed effectively to team goals, they may benefit from training in leadership skills. Furthermore, there is an identified need to promote consistency in leadership training approaches across health professional degrees [25]. Although concerns surround the place of leadership training within crowded healthcare curricula [26], our study suggests that interprofessional learning activities provide an opportunity to frame and embed leadership skills training, practice and assessment for a range of health professional degrees. Interprofessional team learning is increasingly used as a teaching and learning method in health professions education [27]. The interprofessional setting provides the opportunity for faculty to meaningfully address the topic of leadership both in university and clinical practice settings.

Importantly, given the high number of student feedback comments regarded as unconstructive, training in how to provide feedback will likely assist in the growth of students’ leadership skills. A recent study on peer review using a specific rubric to assess the quality of medical student peer feedback during a team exercise highlighted the need for training in this area. Common breaches in professional feedback included ‘cutting and pasting’, as well as banal feedback [28]. This study found that while students were comfortable identifying positive learning behaviours of their peers, they were less able to identify needs for improvement (gap) and detail a plan for improvement (action) [28].

### Limitations

To our knowledge, this study is one of the first qualitative studies to explore the leadership qualities of health professional students identified by team members during a large scale interprofessional learning activity. Findings of this study may not be generalisable to other educational settings.

## Conclusion

Leadership involves influencing team members in a process towards achievement of a common goal [14]. Effective leadership is a complex and highly valued component of clinical practice, where changes in healthcare systems occur rapidly. Our study shows that although most students demonstrated a reasonable ability to display leadership behaviours appropriate to teamwork, further development is needed through training. Leadership skills are an expectation of health professional graduates, and should be explicitly taught and vertically integrated within interprofessional education curricula. Further research is warranted in how students contribute to and understand the requirements of leadership within interprofessional teams.

## Data Availability

Datasets supporting the conclusions of this article are included within the article. Additional data at the level of individual students is not available as per confidentiality agreements approved by the Human Research Ethics Committee, University of Sydney.
